# Effects of Personality Traits Concerning Media Use Decisions on Fear of Missing Out and Social Media Use Behavior

**DOI:** 10.3390/bs12110460

**Published:** 2022-11-18

**Authors:** Sheng-Cheng Lin, Er-Ting Jian

**Affiliations:** Department of Information Management, Tunghai University, Taichung 407227, Taiwan

**Keywords:** maximizing tendency, regret tendency, social comparison orientation, fear of missing out, compulsive use, surveillance use, technostress

## Abstract

We could view the phenomenon of fear of missing out (FoMO) as a dilemma of too many choices about social media. Although there are already various studies on FoMO, there is still a lack of studies on what personality traits concerning media use decisions will contribute to FoMO or how FoMO mediates these personality traits and people’s social media use behavior, and, thus, corresponding negative emotions. This study explored the causes of FoMO in a FoMO moderated mediation model using maximizing tendency before the choice was made, social comparison orientation when making choices, and regrets tendency after the choice was made. The results showed that (1) there is a non-significant influence between maximizing tendency and FoMO, (2) regret tendency is a positive influence on FoMO, (3) social comparison orientation is a positive influence on FoMO, (4) FoMO is a positive influence on the compulsive use of social media and surveillance use of social media, (5) FoMO exhibited a full mediating effect on the relationship between regret tendency and social media surveillance use, (6) FoMO exhibited a full mediating effect on the relationship between social comparison orientation and social media compulsive use.

## 1. Introduction

Fear of missing out (FoMO) [1] is a psychology of restlessness where a person fears that others might be having meaningful experiences from which he or she is absent. Research discussing FoMO is increasingly popular [2]. Tandon et al. [2] conducted a meta research study, which synthesized 58 empirical studies addressing FoMO. They suggested that future studies should focus on exploring and examining other possible antecedents of FoMO, and possible variables that may be examined could include personality traits. Reer et al. [3] also contended that individual differences in psychological traits contribute to the development of FoMO. Moreover, in addition to examining the antecedents and consequences, they also identified that relatively few empirical studies have examined the mediating influence of FoMO. Therefore, they suggested scholars may investigate psychological influencers, such as the mediating effects of causes due to which individuals choose a specific social media platform and the feelings they might derive from its usage. These gaps in the literature led to the motivation for this study.

We may say that the phenomenon of FoMO is a dilemma of too many choices. People may choose whether to get connected or not and which social media (e.g., Facebook, TikTok, Instagram, various virtual communities) to participate in if they only have a little time. There are three possible situations, namely: fear of the possibility of missing out on something before the choice is made; fear of making a wrong choice when an individual often pays attention to information about others extensively and compares oneself to others to decrease his or her sense of uncertainty; and fear of having missed out on something already, after the choice is made. This study explored the causes of FoMO in a FoMO moderated mediation model using maximizing tendency before the choice was made, social comparison orientation when making choices, and regrets tendency after the choice was made. We could confirm no similar studies addressing this issue existed due to Tandon et al. [2]’s latest review of FoMO-related works. Furthermore, the fast flow of information in social media intensifies people’s FoMO, which leads to a situation where people seek more connections with social media so that they can keep in touch with others and with all kinds of information; this is similar to a compulsive behavior. When the compulsive behavior cannot be resisted, negative consequences related to emotional disturbance, such as melancholia and stress, occur [4]. As one characteristic of FoMO is the eagerness to know what others are busy with, namely, to give continuous attention to others, FoMO is like a kind of surveillance behavior. One of the main characteristics of social media is that it offers opportunities for people to monitor each other using social media. Therefore, this study added compulsive use of social media and social media surveillance into the FoMO moderated mediation model as types of social media engagement.

Based on this research background, the purposes of this study were as follows: (1) to investigate whether three personality traits: maximizing tendency, regret tendency, and social comparison orientation have positive influences on FoMO, respectively; (2) to examine whether FoMO is a positive influence on compulsive use and surveillance use of social media; (3) to explore whether compulsive use and surveillance use of social media are positive influences on technostress and the negative feelings of social comparison, respectively; and (4) to examine whether FoMO exhibits mediating effects on the relationship between three personality traits and compulsive use and surveillance use of social media. The first research objective answered the call of Tandon et al. [2] to examine possible antecedents of FoMO, such as personality traits. The last research goal aimed to fill up the research gap of investigating the mediating effects of causes due to which individuals choose social media and the feelings they derive from its usage.

This paper is organized as follows. Section 2 describes the FoMO moderated mediation model, which serves as the basis of the research model to investigate the relationships between personality traits concerning media use decisions and social media engagement behavior, and then Section 3 describes the proposed research model and related hypotheses. The research method is elaborated on in Section 4, and research data are analyzed in Section 5. Given the study’s results, further discussion and implications for managing the condition are proposed in Section 6.

## 2. Fear of Missing Out (FoMO) Moderated Mediation Model

Przybylski et al. [1] defined FoMO as a psychology of restlessness where a person fears that others have obtained more meaningful experiences but he or she is unable to share in them. One characteristic of FoMO is the eagerness to know what others are busy doing. FoMO is seen as a form of social anxiety, for social media information causes attention toward some social opportunity and the fear that someone might miss out some novel experience or other more satisfactory situation [5].

Przybylski et al. [1] further conducted an in-depth study on the related factors of motivation, behavior, and sense of happiness based on self-determination theory. They developed a quantitative Fear of Missing Out Scale (FoMOs) to evaluate FoMO. The results indicated that FoMO can be used as a mediating factor between psychological satisfaction and social media engagement. Relatively lower psychological satisfaction is correlated to relatively higher FoMO, and FoMO is shown to have a close relationship with social media engagement. Participants with high FoMO have more social media engagement behavior and FoMO acts as the driving force behind social media. Przybylski et al. [1] also explored the relationship between FoMO and a sense of happiness. The results showed that FoMO is negatively related to emotions and life satisfaction. People with high FoMO tend to more frequently check social media before they sleep, or get up, or have dinner, and they are more easily distracted by the temptation of social media during class time and while driving.

Based on the FoMO moderated mediation model proposed by Przybylski et al. [1], Alt [6] explored the correlation between three learning motivations and the social media engagement of college students during class time. He found FoMO as the mediating factor for the positive correlation between the external world and a non-learning motivation, and social media engagement. Thus, the study of Przybylski et al. [1] provides a good foundation for understanding FoMO and how it influences people’s psychological health. There are many studies that adopted the FoMO moderated mediation model to conduct research. For example, Wang et al. [7] used it to test the relation between the need to belong and authentic self-presentation on SNSs, and indicated that FoMO mediated the association between the need to belong and adolescent authentic self-presentation on SNSs. Dou et al. [8] employed the FoMO moderated mediation model to explore the relationship between perceived social support and FoMO. They concluded that perceived social support was negatively associated with FoMO, and psychological needs partially mediated the relationship between perceived social support and FoMO. Bodhi [9] examined the association between work-related social media use, psychological well-being, and innovative work performance and explored the mediating role of psychological well-being and the moderating role of FoMO. They found that FoMO moderates the indirect relationship between work-related social media use and innovative work performance. Li and Ye [10] also adopted the FoMO moderated mediation model to investigate the relationship between FoMO and irrational procrastination in a mobile social media environment and the mediating role of cognitive failure. They found that FoMO positively predicted irrational procrastination in the mobile social media environment, and cognitive failure had a complete mediating effect on the relationship between FoMO and irrational procrastination. The present study elaborates on three personality traits concerning media use decisions: maximizing tendency, regret tendency, and social comparison orientation and possible relationships with FoMO in the next section.

## 3. Hypotheses

### 3.1. Maximizing Tendency and FoMO

Schwartz et al. [11] proposed the concepts of maximizing tendency and satisficing. Maximizing tendency occurs when someone faces many choices; he or she will try his or her best to collect and analyze information to find the optimal situation. Satisficing is when someone does not like to spend too much time and thus can accept an option that reaches his or her standard of acceptability. To measure individual differences, Schwartz et al. [11] developed a set of maximizing tendency scales. They pointed out that maximizers pursue interest maximization when they are faced with multiple choices, and will try their best to gather and analyze information to find the best option. However, in the end, maximizers are rarely satisfied with their own choices because they are also very sensitive to what they will lose because of their choices. Maximizers still think that there are better options even when their choices are not so bad. Chowdhury et al. [12] found that maximizers tend to change their original choices when possible. Maximizers also tend to be dissatisfied with their choices, as other studies have found [13]. Compared with satisficers, maximizers intend to pursue optimal results, leading to more negative emotions and worse experiences [14].

There are countless new pieces of information emerging on social media every minute and we are unable to absorb and process the vast information contained by all possible choices when facing massive and rich real-time information. Maximizers must pay a large amount of time and attention in pursuit of their best interests and they cannot absorb all information in the face of the excessive choices provided by social media because of limited time and attention. Therefore, maximizers always think there are better choices available, are sensitive to what they lose, and will worry about missing something better. Thus, this study assumed that a high maximizing tendency is positively correlated with high FoMO.

**[H1]**  Maximizing tendency has positive influence on FoMO.

### 3.2. Regret Tendency and FoMO

Regret is a negative emotion in cognition and occurs when an individual realizes that if he or she had made a different decision, a better result would have been the consequence [15]. Sagi and Friedland [16] believed that regret occurs when a person finds that the choice he or she has made is not the best one, or there is a gap between the unselected best choice and his or her own choice. It is apparent from this that regret, unlike other negative feelings, will be experienced only after a choice has been made.

Dan Ariely indicated that FoMO depends on the concept of regret; people may imagine “I could have done that” because they are afraid of making wrong decisions on how to use time [17]. Social media operate in real time, which is entirely different from talking about what happened last week. Various kinds of real-time information and activities are constantly reminding us of what others are doing, what is happening right now, and what we have missed, thus intensifying the imagining of “what I could have done.” As people have distinct lives and livelihoods at the same time, and there is no limit to the comparisons that can be made, people always miss something because they cannot absorb and follow the latest information on social media where information updates rapidly. Therefore, people who often experience regret will imagine that things could have been different, thus intensifying the fear of missing out and making them feel that they have failed to do something meaningful, while others engage in meaningful activities all the time. Therefore, this study assumed that a high regret tendency is positively correlated with a high FoMO.

**[H2]**  Regret tendency has a positive influence on FoMO.

### 3.3. Social Comparison Orientation and FoMO

People naturally have comparison needs, and when receiving information from others, they unconsciously make comparisons [18]. Mussweiler et al. [19] indicated that when faced with information about what others have realized or not, people will connect this information to themselves, which means that social comparison occurs almost all the time in our daily life as people effortlessly acquire others’ information through a variety of means. Wood [20] also considered that social comparison is an interactive process prevailing in daily life. Not everyone likes to conduct social comparison with others, and the degree and frequency of comparison varies. People with a high tendency to make social comparisons are more interested in others’ performances [21].

As an easily available medium containing massive information, social media are used by those with high social comparison orientations to seek information and find out information about the performance of other people. Yet people will have the feeling of missing something because they will inevitably find out about something they do not have that other people do. Therefore, this study assumed that a high social comparison orientation is positively associated with a high FoMO.

**[H3]**  Social comparison orientation has a positive influence on FoMO.

### 3.4. FoMO and Social Media Use

O’Guinn and Faber [22] defined compulsive behavior as the lack of capacity to control instinctive needs or desires in terms of responding to the feelings or activities involved in achieving, using, and experiencing; this leads to individuals’ repeating behaviors and, finally, does harm to individuals themselves or to others. Its main feature is repetitive and meaningless behavior patterns [23]. Compulsive behavior can be widely observed among ordinary people. Driven by anxiety, people repeat some behaviors to seek peace of mind. FoMO is considered a type of social anxiety. Prior literature has concluded that FoMO is linked to many psychopathological symptoms, such as anxiety [24], depression [25], diminished well-being [26], and social media fatigue [27]. Information on social media often makes people force themselves to focus on the opportunities of social contact that they might miss, including some fresh experiences or other satisfying circumstances [5]. The problem for people with high FoMO is that they observe things that other people work on or have, which they do not; thus, they are more likely to use social media to stay in touch with other people or to acquire various information.

FoMO’s association with excessive use of digital technology can lead to a permanent state of being online [28,29]. The strong desire to stay in touch is potentially dangerous for the reason that it can incite people to check their mobile devices [30]. Through studies on smartphones, Oulasvirta et al. [31] found that the quick access and response that smartphones offer can trick people into checking their phones—a compulsive behavior—more frequently. Compulsive behavior was defined as being unable to control an instinctive need and a desire to respond to information about the feelings or activities that are obtained, used, or experienced. Tandon et al. [32] examined the association of FoMO and phubbing. The results indicated that FoMO has a positive association with phubbing. High FoMO can drive people to seek connections through social media, and consequently increases behavior such as repeated checking. Therefore, this study assumed that high FoMO is positively associated with high compulsive use of social media.

**[H4]**  The degree of FoMO has a positive influence on the compulsive use of social media.

Surveillance is the persistent attention to gathering data and information about other people deliberately by electronic means, such as information systems and mobile devices [33,34]. Social media have become ideal surveillance tools because of their characteristics of providing abundant information that is easy to access and save, without geographic restrictions [35]. Tandoc et al. [36] found that the specific purposes of Facebook, including tracking the posts and activities of other people, checking photos, and skimming through other people’s updates, could be described as surveillance. As one characteristic of FoMO lies in the desire to know what others are doing at any given time, people with high FoMO use social media for the purpose of staying in touch with other people and paying attention to the activities and information of their friends [5]; thus, much persistent attention to other people resembles surveillance behavior. This study thus assumed that high FoMO is positively associated with high surveillance use of social media.

**[H5]**  The degree of FoMO has a positive influence on the surveillance use of social media.

### 3.5. Social Media Use and Negative Feelings

Technostress is a modern disease caused by being unable to cope with new computer technology in a healthy way [37]. Tarafdar et al. [38] developed and verified factors that caused technostress and referred to them as “technostress creators.” They listed five types of factors that specify the situations where technostress results from the use of ICT, but only two factors are related to this study: (1) Techno-overload: assistance tools, such as mobile technology, applications, and software require employees to handle either external or internal information influx, which leads to an overload of communication and information. Employees are then faced with too much information that exceeds their abilities to cope and to use efficiently [39]; this will result in stress. In other words, ICT users are forced to work faster and longer. (2) Techno-invasion: everyday work is prolonged by the continuous capacity for connection. Employees use e-mails or mobile devices to make connections any time and any place. They frequently feel forced to reply and, to some extent, feel restless without connections. Owing to this continuous capacity for connection, employees feel that they are unfree, and their personal time and space are invaded by these technologies, which again leads to stress. In other words, the pervasiveness of ICTs invades the personal lives of employees.

In their studies on the influence of psychological pressure on health in daily life, Charles et al. [40] pointed out that the everyday contact with pressure sources has long-term negative influences on the psychological health of users. Lee et al. [41] found that the compulsive use of smartphones causes higher technostress in users. The overload of technology brings about pressure by providing too much information, and because technological intrusion has the capacity to connect people continuously, their private life comes under pressure [38]. Given the capacity in recent years to connect with social media and mobile technology at any time, and because of the rich information it provides, users must process excess information rapidly; they will thus be under technostress both because of being overloaded and because of the technology’s influence on daily life (technology intrusion). Chai et al. [42] found FoMO to moderate the effect of social overload on social media use and well-being. Apaolaza et al. [43] also confirmed that compulsive mobile SNS use induces stress. Rautela and Sharma [44] showed that compulsive use significantly and positively impacts mental and psychological health. Therefore, this study assumed that high compulsive use of social media is positively associated with a high technology overload and the technological intrusion of social media.

**[H6]**  Compulsive use of social media has a positive influence on technology overload.

**[H7]**  Compulsive use of social media has a positive influence on technology intrusion.

Social media allows users to engage in impression management through various kinds of technology to optimize their self-presentation and promote expected relationships. Goffman [45] believed that self-presentation is a form of impression management, which puts emphasis on the presenting behavior in order to “guide” others during interactions, just as a performer on stage does when trying to get recognized in a certain light by the audience. Almost all the information about other people obtained and accessed on social media involves constructed impressions, in which individuals try to present an attractive picture, and the comparison between these beautified impressions and one’s current condition may bring about negative feelings. Compulsive social media users may renew a series of close-up selfies at a top-grade restaurant repeatedly to create impressions, but soon induce negative feelings when compared with the real situation. Aladwani and Almarzouq [46] indicated compulsive social media use arises due to self-awareness factors, and thus may predict problematic outcomes. Excessive use of social media will cause many different comparison behaviors and negative effects; thus, this study assumed that high compulsive use of social media is positively associated with the negative feelings involved in social comparisons.

**[H8]**  Compulsive use of social media has a positive influence on the negative feelings of social comparisons.

Surveillance use is a persistent and deliberate attention to gathering data and information about other people by electronic means [33,34]. According to Tarafdar et al. [38], technology overload can impose pressure through providing too much information and technological intrusion, because of its capacity to connect continuously, which puts private life under pressure. As rich information exists on social media, users will suffer from technostress by being overwhelmed (technology overload) and from its influence on daily life (technology intrusion) because they can obtain extensive data and information about other people using frequent surveillance. Obtaining information about other people frequently will cause more comparison behaviors [18] and the negative feelings that are a result of social comparison [47,48,49]. Therefore, this study assumed that high surveillance use of social media is positively associated with high technological overload, technological intrusion, and the negative feelings resulting from social comparisons.

**[H9]**  Surveillance use of social media has a positive influence on technology overload.

**[H10]**  Surveillance use of social media has a positive influence on technology intrusion.

**[H11]**  Surveillance use of social media has a positive influence on the negative feelings of social comparisons.

**Figure 1:**
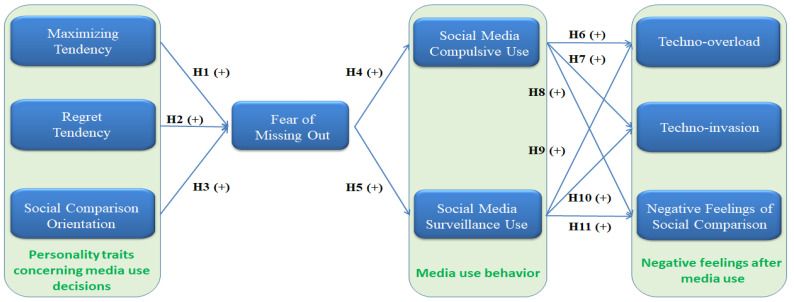
Research Model of Present Study.

Figure 1 depicts the research model for this project, which is based on the assumptions listed above:

## 4. Methods

### 4.1. Questionnaire Design

This study employed a questionnaire to avoid measurement errors; all the questions in it were adopted from scales that were already verified in the literature, as shown in Table 1. The original questions were modified according to the aims of this study but without changing their original content. A seven-point Likert scale was adopted for all dimensions ranging from 1 (strongly disagree) to 7 (strongly agree).

### 4.2. Questionnaire Distribution

The online questionnaires in this study were completed with the Google online form design and were distributed through direct transmission and through links; participants could begin filling in the questions by clicking on the links. Results were received on completion through Google Cloud Drive, the questionnaire collection allowed for checking in real time, and the overall operation interface reduced the time involved. The sample format was chosen to allow for export and data analysis after the questionnaires were collected. Before the questionnaires were officially distributed, there were 20 participants who conducted pre-tests and provided their suggestions for revision to ensure the questions’ meanings and expression were clear and readable. The pre-test results did not delete any question but only revised the meanings and expressions slightly. Since this study primarily discussed phenomena caused by social media, the respondents involved were all users of social media. Therefore, the present study adopted convenience sampling to collect data. To increase the generality of the research study, the online questionnaires were distributed through multiple sources, such as various famous virtual communities in Taiwan, such as PTT (a virtual community of graduate and college students), BabyHome (a virtual community for parenting), Facebook, and Bahamut (a virtual community of game players). Data were collected from 21 May to 1 July 2021, using a 48-question online survey (7 demographic, 41 content questions).

### 4.3. Tool for Data Analysis

This study used partial least squares (PLS) as a statistical tool for data analysis, including the analysis validity and the verification of the study models. PLS is an analysis tool of structural equation modeling (SEM) and an analysis technology for exploring or establishing prediction models, which are superior to common linear structural relations models (LISREL), especially for the analysis of causal models of potential variables. PLS has several advantages: it does not require too many samples; there are no requirements for the statistical distribution of data; and there are few limitations for measurement scales. It can overcome the collinearity of multiple variables, predict exploratory analysis, and process questionnaire scales of reflective and formative indexes. For these reasons, this study adopted PLS as the method for the analysis of the data. This study conducted analysis with the software SmartPLS 4 developed by Ringle, Wende, and Will [55] and calculated the significance of model coefficients by selecting 5000 samples randomly using the bootstrapping algorithm. In addition, we adopted a software named jamovi to calculate McDonalds’ ω coefficient of reliability [56]. We also used SPSS 19 to examine common method variance (CMV).

## 5. Data Analyses

### 5.1. Sample Characteristics

This study collected data from 348 respondents. After excluding the invalid respondents, 310 valid respondents remained, giving a qualification rate of 89%. The descriptive statistics of the sample are presented in Table 2. There were more male respondents (177; 57.1%) than female respondents (133; 42.9%). Most of the respondents were aged from 21 to 30 (65.2%), suggesting that there were more young people than old involved in the survey. The distributions are akin to the census data of Digital 2021 Taiwan [57]. As for educational background, most respondents had graduated from universities and colleges (224; 72.3%) or graduate schools (67; 21.6%). Although most were students (190; 61.3%), there were 120 respondents without higher education (38.7%).

### 5.2. Reliability and Validity Analysis

McDonalds’ ω coefficient provides more realistic estimates of true reliability of scale than Cronbach’s Alpha [58]. This study employed it to test the reliability of the questionnaire and examine the internal consistency of the questions designed for each dimension. The reliability analysis results are exhibited in Table 3. The McDonalds’ ω coefficients for all the dimensions of this study were above 0.7, indicating a high level of internal consistency.

In addition, SmartPLS 4.0 was applied to test the measurements for validity, including convergent and discriminant validity. The items with low factor loading values were removed from the final questionnaire, including MT5, RT4, SCO4, SCO6, SMSU1, NCF3, and TOV4. The factor loadings of all the remaining questions were greater than 0.5. The validity analysis after adjustment is also shown in Table 3. All the results of the factor loadings, composite reliability (CR), and average variance extracted (AVE) analyses exceeded the threshold value of the criteria, indicating that the dimensions used in this study had a certain convergent validity.

The correlation analysis results of all the dimensions are presented in Table 4. The square root of the AVE of each dimension was greater than its correlation coefficients with other dimensions. Therefore, the dimensions of this study had considerable discriminant validity. Furthermore, we dealt with the problem of common method variance (CMV) by using a Harman’s one-factor test. The maximum variance explained by a single factor in the model is 34.828%, which is lesser than 50% and thus indicates the absence of common method bias.

### 5.3. Test of Hypotheses

**Figure 2:**
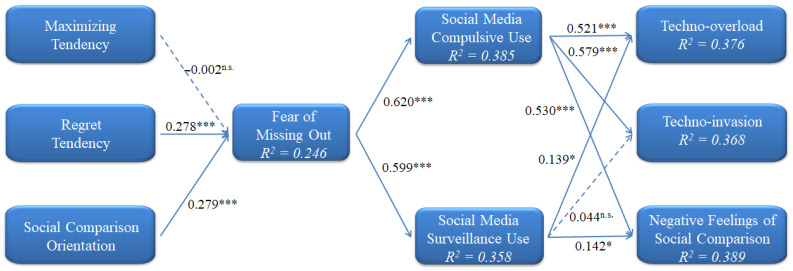
PLS Results of the Model (* *p* < 0.05; *** *p* < 0.001; n.s. not significant).

PLS were used in this study to examine the conceptual model and the corresponding hypotheses. The path coefficient of each dimension is exhibited in Table 5. The results included the significant degree of the path coefficients and the explanatory power of the model (R Square, R^2^). The path coefficients show the degree of relationship among variables, whereas R^2^ represents the predictability of the model over the dependent variables. The explanatory powers of each dependent variable (R^2^) were FoMO (24.6%), SMCU (38.5%), SMSU (35.8%), TOV (37.6%), TI (36.8%), and NCSM (38.9%). The model had a respectable explanatory power over all dependent variables (Figure 2).

### 5.4. FoMO Mediating Effect Testing

We conducted the Sobel Test [59] to examine the mediating effects of FoMO. According to the test results in Table 6, FoMO did not have any mediating effect between maximizing tendency and media use. FoMO partially mediated the relationship between regret tendency and social media compulsive use. FoMO also partially mediated the relationship between social comparison orientation and social media surveillance use. Moreover, FoMO exhibited a full mediating effect on the relationship between regret tendency and social media surveillance use. Finally, FoMO exhibited a full mediating effect on the relationship between social comparison orientation and social media compulsive use.

## 6. Discussions and Conclusions

### 6.1. Discussions

**H1**: It is possible that maximizing tendency and satisficing decision-making strategies are, to some extent, situational; when an individual is confronted with different situations, he/she may adopt different decision-making strategies. Therefore, the maximizing tendency of an individual may have “domain specificity.” In other words, an individual can be a maximizer in one domain and a satisficer in another domain [11]. For example, a person may be demanding and seek constant improvement in his or her work but show less of an impulse to do so in his or her daily life. Similar examples can also be found in shopping habits. For example, a person may spend a great deal of time searching for a desirable keyboard or mouse but make less effort in shopping for clothes. This study focused mainly on the most updated topics, information, or social activities related to social media; the maximizing tendency of the respondents might only show in other fields. As a result, the research could not fully define whether a respondent is a maximizer by examining areas solely related to social media.

There were preset situations in the maximization scale developed by Schwartz et al. [11]—such as watching TV, picking a gift, or renting a movie—which might cause the above-mentioned problem. However, the scale used in this study was the re-defined scale developed by Diab et al. [50] based on trait theory, which considered maximizing tendency a specific trait. Traits are relatively stable habitual patterns, which should remain unchanged even in different situations. Future studies may further verify this assumption.

**H2**: As was suggested by Dan Ariely, professor of psychology and behavioral economics from Duke University, FoMO depends on people’s concept of regret. When people are afraid that they have made wrong decisions about their utilization of time, they might generate a feeling of “I could have been” [17]. The rich and instant feeds and messages on social media and the various real-time information and activities are constant reminders of what others are doing, what is happening, and what we have missed, which exacerbates this feeling. Due to the finiteness of time, it is pointless to compare what one is doing with what others are doing. Since each individual is living his or her own life in his or her own style, differences in what an individual is doing compared with others are inevitable. Moreover, humans have a limited capacity for focus; it is unlikely that a person can fully absorb and keep up with all the fast-flowing, updating information on social media, and some things are likely to be unattended to. For that reason, people with high regret tendency tend to feel that they have not made a good choice or have made a relatively poor choice. This feeling of regret leads to high FoMO. Hence, H2 was accepted.

**H3**: Van der Ze et al. [21] suggested that people with a higher social comparison orientation presented more interest in the ideas and performance of others. As a form of media with easy access to a large volume of information, social media has become an important tool for many people to access and interact with others and to observe what other people have and how they do things. People with high social comparison orientation tend to actively seek information from social media to acquire information about other people and their performance. In addition, Taylor and Lobel [60] described the specific behaviors of social comparison as “paying lots of attention to information about others” and having a “self-evaluation against others.” Since people have limited time and space available, it is likely that, while absorbing the information of other people and comparing with others, they might discover things that other people possess that they do not, which might trigger FoMO. Therefore, H3 was accepted.

**H4**, **H5**: As was mentioned in past research, a characteristic of FoMO caused by the information generated from social media lies in the desire to keep in touch with what others are doing. Individuals with a high degree of FoMO tend to pay too much attention to the things that their friends are doing or possessing, which they themselves are not doing or possessing [1,5]. An overly strong desire to stay in touch has potential danger as it encourages people to look at their mobile devices [30]. For that reason, a high degree of FoMO will drive people to seek a connection to social media and increase the repeated checking behavior. These conclusions are the same as the findings of the study by Przybylski et al. [1]: people with high degrees of FoMO tend to frequently check information on social media before bedtime, after waking up, or during meals and are easily distracted by social media when they are in class or driving. According to the study by Oulasvirta et al. [31] on the use of smartphones, frequent information checking on social media is a compulsive behavior. People who suffer from a high degree of FoMO fear that they might miss out on things if their connection to social media is interrupted. Therefore, regardless of time or location, the compulsive behaviors of constantly connecting and refreshing social media exist. H4 was accepted. Consistent with past studies, people with a high degree of FoMO use social media to keep in touch with others, and they observe the activities of and get information about their friends. On that account, they tend to show more behaviors of social media surveillance use. H5 was accepted.

**H6**, **H7**: Corresponding to past studies, failing to resist compulsive behaviors can lead to negative consequences, including psychological distress, such as depression and stress. Moreover, uninterrupted use of technology will also exacerbate psychological distress [4]. Since seeking connection with social media requires a PC or mobile devices, *technostress* can be used as an effective indicator of psychological distress caused by the compulsive use of such ICT devices [37,61]. For example, the compulsive use of smartphones can lead to higher technostress in users [41].

Tarafdar et al. [38] pointed out that techno-overload refers to the overload of communication and information caused by the usage of technology. Assisting tools and software, such as mobile technology and apps, have enabled users to handle the influx of information from external or internal sources and expose them to more information than they can effectively deal with and make use of [39]. As a result, they create a feeling of stress. The compulsive use of social media makes users feel that they have to keep up with the updating speed of social media messages by receiving and processing excessive amounts of information, leading to pressure caused by overburden. Therefore, H6 was accepted.

According to Tarafdar et al. [38], techno-invasion refers to the situation in which, due to its feature of continuous and easy access, technology has made boundless connection and communication possible. However, people often feel like they are forced to respond to others or are uneasy when not connecting to someone for a certain period. This continuous connection makes employees feel that they no longer have any freedom, as their private time and space have been invaded by such technologies, thus creating pressure. Compulsive use of social media always means non-stop connection with social media and places, which will eventually cost the individual time, affect his or her normal life, and make him or her feel that technology has invaded his or her life. Thus, H7 was accepted.

**H8**: When comparing themselves with others, people tend to generate positive or negative feelings [47,48]. The rich information supplied by social media, including the personal information about other members, provides many opportunities for people to compare themselves with each other. However, such computer-mediated communication tools also provide users with a variety of technologies for impression management, so they can optimize their self-presentation and enhance their intended relationships. As a result, most of the information acquired from social media tends to be modified and beautified to give a good impression. When comparing these beautified impressions with one’s actual, non-beautified situation, a person may generate negative feelings caused by the difference.

Accordingly, past studies on social comparison on social networks, such as Facebook, found that excessive use of Facebook will lead to more behaviors of social comparison [49] as well as negative impacts. The frequency of social comparison on Facebook is positively correlated with negative feelings. For example, negatively comparing oneself with others might cause depressive symptoms [62]. Frequent social comparison on Facebook may make users feel unhappy or unworthy and may bring feelings of envy, leading to stress for the users [63,64]. Yang et al. [65] conducted a meta-analysis to investigate the effect of Facebook social comparison on an individual’s well-being. They indicated a negative association between Facebook social comparison and well-being. Excessive use of social media may trigger many comparative behaviors, leading to negative impacts. H8 was accepted.

**H9**, **H10**, **H11**: Surveillance is defined as purposely collecting data and information about other people through electronic means, including information systems or mobile devices, and a continuous behavior of close following [33,34]. Surveillance use refers to the use of specific means to follow others, including tracking their posts and activities and viewing their photos and status updates to keep up with their current situation and obtain information. When a person invests his or her time and attention to observe others, he or she is likely to be overloaded with an excessive amount of information through frequent surveillance behaviors, since social media is filled with various instant information. Moreover, due to the feature of instance, users feel that they must process this excessive information faster. This is in line with Tarafdar et al.’s [38] definition of techno-overload as the technostress caused by overloading of information. Hence, H9 was accepted. Corresponding to past studies, frequently acquiring large amounts of information about others will often lead to more behaviors of social comparison [18] and cause negative feelings after making comparisons [47,48,49]. H11 was accepted.

The results of this study showed no significant correlation between frequent surveillance use and techno-invasion. Surveillance use does not invade the user’s personal life (it is, in fact, the people under surveillance who are invaded). In addition, although frequent surveillance use might also result in continuous connection with others, it does not require response or forced communication since the user can simply be an observer (commonly known as “lurking”). Even though frequent surveillance use might equally cost the user a significant amount of time, its impact on the user is different from that of compulsive surveillance use, as the latter refers to a situation in which a user cannot control his or her behavior. Since the measurement of surveillance use adopted by this study cannot detect whether the user can control his or her surveillance use behavior, H10 was rejected.

Noticeably, FoMO exhibited a full mediating effect on the relationship between regret tendency and social media surveillance use. For people with greater regret tendency, once missing out happens, they will regret not being connected earlier. Therefore, to prevent missing out, they will continually monitor what is going on, and thus contribute to surveillance use. This could eventually result in negative feedback and thus make them unhappy. Cho et al. [66] also seeks to understand the regret behind social media use. They investigated when and why people regret using different features of social media. They identified three patterns of features with different characteristics that lead to regretful use. Based on the research result, we should pay more attention to the important roles of regret and figure out how social media can be designed to reduce regretful use. After all, we use social media for leisure and fun.

Moreover, FoMO also demonstrated a full mediating effect on the relationship between social comparison orientation and social media compulsive use. Similarly, if someone was more social comparison oriented, to avoid missing out, he or she would always keep online to collect information about others extensively and compare themselves to others to decrease his or her sense of uncertainty, which would thus result in compulsive use. FoMO could make people uneasy; we should remind them that it is social comparison tendency instead of FoMO per se that makes people unhappy, along with regret tendency. For people who have both regret and social comparison tendencies, we would suggest that they use social media just for liaison, but not get involved, based on the research results, or place emphasis on the joy of missing out (JoMO), since Aitamurto et al. [67] indicated users can experience both FoMO and JoMO at the same time. Their findings suggested that FoMO and JoMO can be two integral qualities and that FoMO may not be as negative a factor as previously thought. JOMO is a sociological phenomenon that is a response to FoMO. As was suggested by an entrepreneur, Anil Dash, people may enjoy what they are doing in each moment without worrying about what everyone else is doing. Since regret tendency and social comparison orientation are some sort of personality traits, and we know it is not easy to change personality traits, it seems JoMO could be a possible solution for them. It is important to realize that the cause of this problem is our minds and is embedded within human nature rather than technology, and we should be able to break away from the shackles of regret. We should learn how to experience JoMO by focusing on the things that are important to us and savoring the moment with what we currently possess. We should enjoy the current moment and our own choices and understand that other options are not better; they are just different. Certainly, to achieve this outcome might not be easy; however, we may want to consider why it is that we regret something, why we are afraid of missing out, or whether we are actually “missing out” at all. Through thorough reflections upon these questions, we might discover things that have real significance to our wellbeing.

In conclusion, Tandon et al. [2] reviewed several FoMO-related studies and concluded that there is a need to study the mediating effect of two factors—psychological and usage—on the associations between individuals’ use of platforms, the experience of FoMO, and subsequent outcomes. This present study tried to answer the call and investigate three personal inclinations concerning media use decisions on FoMO and media use behaviors and corresponding feelings. According to the results, maximizing tendency was not salient on FoMO; whereas social comparison orientation and regrets tendency both played important roles on FoMO. FoMO demonstrated a full mediating effect on the relationship between regret tendency and social media surveillance use. Furthermore, FoMO also revealed a full mediating effect on the relationship between social comparison orientation and social media compulsive use.

### 6.2. Managerial Contribution

This study aimed to explore the cause and impact of FoMO in the setting of social media. The results showed that regret tendency and social comparison orientation had a significantly positive correlation with FoMO. Moreover, FoMO was significantly positively correlated with the behaviors of compulsive use and surveillance use of social media, further leading to technostress. To maintain long-term operation, a social media site needs to retain its users and make sure the users make return visits. The findings of this study can inspire social media operators to utilize users’ FoMO. The following measures are suggested. (1) Ensure information is new and updated, and make users feel that the site is worth their frequent attention, otherwise leading to a feeling of regret when they miss relevant information. This also means that operators should try to avoid reposting the same messages or excessively posting similar information. Repeated messages tend to annoy users, while unrefreshed information is not considered valuable to users. As a result, users might cast their attention elsewhere. (2) Provide substantial and original information. Regardless of their interests or whether seeking comparative information for shopping reference, asking for other people’s opinions, or discussing certain topics with others, users tend to go to places with enough and original information. Meaningless posts and copied or reposted information from other resources will often demotivate users to pay attention and participate in the site. (3) Create trends and fads that attract individuals through groups by stimulating users in a way that creates the need to keep up with others, and urge them not to fall behind.

### 6.3. Limitations

This study employed a self-report questionnaire to collect data. Therefore, some of the answers might not truly reflect the actual situation of the respondents. In other words, some respondents might refuse to admit that they have certain tendencies or feelings or might assume they have never experienced the described situations when answering the questions. Hence, the results of the study might be affected.

The sample of the study mainly focused on users from famous virtual communities in Taiwan. Although there was a certain number of respondents other than high school students, most respondents were still young. Since almost the entire population of Taiwan uses social media, our sample may not be representative. Moreover, there is a possibility that some respondents completed more than one questionnaire.

### 6.4. Suggestions for Future Research

This study did not measure maximizing tendency based on different fields. Further studies can measure maximizing tendency targeting specific fields. There are various types of social media; each has its own preference. For example, different social media sites have different social activities and user information; different categories of Internet forums have different events, news, information, or interesting reports; and social media such as LINE provide a lot of real-time messages from friends, elders, and supervisors as well as discount information from official brand accounts. Focusing on a specific type of social media can improve the accuracy of the measurement and generate more targeted results.

Future studies can define the FoMO scale and separate it into two dimensions: the fear of happenings that he or she has missed out on and the fear of the possibility of missing out on something in the future. Then, researchers can explore the causes and impacts of each dimension.

## Figures and Tables

**Table 1:** Questionnaire Design.

Variables		Scales	Sources	Items
Maximizing Tendency		Maximizing Tendency Scale	Diab et al. [50]	5
Regret Tendency		Regret Scale	Schwartz et al. [11]	4
Social Comparison Orientation		Iowa-Netherlands Comparison Orientation Measure	Gibbons and Buunk [51]	6
Fear of Missing Out		Fear of Missing Out Scale	Przybylski et al. [1]	6
Compulsive Use of Social Media		Compulsive Internet Use Scale Compulsive Usage of Mobile Phones Scale	Meerkerk et al. [52] Lee et al. [41]	6
Surveillance Use of Social Media		Surveillance Use Scale	Tandoc et al. [36]	4
Techno-stress of Social Media	Technology Overload	Tarafdar Technostress Scale Compulsive Usage of Mobile Phones Scale	Tarafdar et al. [38] Lee et al. [41]	4
	Technology Intrusion	Tarafdar Technostress Scale Compulsive Usage of Mobile Phones Scale	Tarafdar et al. [38] Lee et al. [41]	2
Negative Feelings of Social Comparison		Affective Consequences of Social Comparison Scale Dispositional Envy Scale	Buunk et al. [53] Smith et al. [54]	4

**Table 2:** Sample Characteristics and Descriptive Statistics.

	Category	Sample Number	Percentage
Gender	Male	177	42.9%
	Female	133	57.1%
	Total	310	100%
Age	Below 20	63	20.3%
	21~30	202	65.2%
	31~40	41	13.2%
	41~50	2	0.6%
	Above 51	2	0.6%
	Total	310	100%
Education	Junior High School or Below	4	1.3%
	Senior High/ Vocational School	15	4.8%
	Graduate School or Above	67	21.6%
	University/College	224	72.3%
	Total	310	100%
Occupation	Student	190	61.3%
	Public Service (Soldiers, Police, Civil Servants, and Teachers)	18	5.8%
	Manufacture Industry	23	7.4%
	Service Industry	37	11.9%
	Technology Industry	18	5.8%
	Finance/Insurance Industry	5	1.6%
	Realty/Real Estate Industry	4	1.3%
	Retail/Wholesale	4	1.3%
	Others	11	3.6%
	Total	310	100%

**Table 3:** Results of Reliability and Validity Analysis.

	Item Code	Standardized Factor Loadings (>0.5)	McDonald’s ω	CR (>0.7)	AVE (>0.5)
Maximizing Tendency (MT)	MT1	0.776	0.733	0.826	0.543
	MT2	0.753			
	MT3	0.677			
	MT4	0.738			
Regret Tendency (RT)	RT1	0.889	0.808	0.881	0.712
	RT2	0.847			
	RT3	0.793			
Social Comparison Orientation (SCO)	SCO1	0.847	0.832	0.884	0.657
	SCO2	0.710			
	SCO3	0.869			
	SCO5	0.805			
Fear of Missing Out (FoMO)	FoMO1	0.738	0.886	0.913	0.637
	FoMO2	0.803			
	FoMO3	0.748			
	FoMO4	0.850			
	FoMO5	0.807			
	FoMO6	0.835			
Social Media Compulsive Use (SMCU)	SMCU1	0.867	0.901	0.923	0.668
	SMCU2	0.893			
	SMCU3	0.810			
	SMCU4	0.768			
	SMCU5	0.808			
	SMCU6	0.748			
Social Media Surveillance Use (SMSU)	SMSU2	0.948	0.957	0.972	0.920
	SMSU3	0.959			
	SMSU4	0.971			
Techno-overload (TOV)	TOV1	0.871	0.878	0.921	0.796
	TOV2	0.928			
	TOV3	0.876			
Techno-invasion (TI)	TI1	0.924	0.790	0.905	0.826
	TI2	0.894			
Negative Feelings of Social Comparison (NCSM)	NCSM1	0.938	0.897	0.933	0.823
	NCSM2	0.937			
	NCSM4	0.844			

**Table 4:** Results of Correlation Analysis.

	FoMO	MT	NCSM	RT	SCO	SMCU	SMSU	TI	TOV
FoMO	**0.798**								
MT	0.229	**0.737**							
NCSM	0.680	0.184	**0.907**						
RT	0.443	0.413	0.419	**0.844**					
SCO	0.442	0.418	0.461	0.593	**0.811**				
SMCU	0.620	0.185	0.613	0.366	0.306	**0.817**			
SMSU	0.598	0.243	0.453	0.316	0.423	0.586	**0.959**		
TI	0.531	0.189	0.560	0.297	0.309	0.605	0.384	**0.909**	
TOV	0.539	0.251	0.553	0.338	0.383	0.603	0.444	0.643	**0.892**

Remark: 1. MT = maximizing tendency; RT = regret tendency; SCO = social comparison orientation; FoMO = fear of missing out; SMCU = social media compulsive use; SMSU = social media surveillance use; TOV = techno-overload; TI = techno-invasion; NCSM = negative feelings of social comparison. 2. The bold numbers in the diagonal are the square roots of AVEs. 3. The non-diagonal numbers are the correlation coefficients between each dimension.

**Table 5:** Path Analysis of the Model.

	Hypotheses	Standardized Path Coefficient	Standard Deviation	*t*-Value
H1	MT → FoMO	−0.002	0.062	0.037 ^n.s.^
H2	RT → FoMO	0.279	0.070	3.962 ***
H3	SCO → FoMO	0.278	0.064	4.322 ***
H4	FoMO → SMCU	0.620	0.037	16.993 ***
H5	FoMO → SMSU	0.599	0.041	14.356 ***
H6	SMCU → TOV	0.521	0.050	10.482 ***
H7	SMCU → TI	0.579	0.050	11.676 ***
H8	SMCU → NCSM	0.530	0.055	9.651 ***
H9	SMSU → TOV	0.139	0.067	2.089 *
H10	SMSU → TI	0.044	0.060	0.741 ^n.s.^
H11	SMSU → NCSM	0.142	0.068	2.105 *

Remark: MT = maximizing tendency; RT = regret tendency; SCO = social comparison orientation; FoMO = fear of missing out; SMCU = social media compulsive use; SMSU = social media surveillance use; TOV = techno-overload; TI = techno-invasion; NCSM = negative feelings of social comparison. * *p* < 0.05; *** *p* < 0.001; n.s. not significant.

**Table 6:** Results of FoMO Mediating Effect Testing.

	Original Sample (O)	Standard Error	Sobel Z (>1.96)	Path Coefficient (*t* Value > 1.96)	FoMO Mediating Effect
MT → FoMO	−0.002 (a)	0.062 (SE_a_)	0.032 ^n.s.^		No mediation
FoMO → SMCU	0.620 (b)	0.037 (SE_b_)			
MT → SMCU				0.180 ^n.s.^ (*t* = 1.341)	
MT → FoMO	−0.002 (a)	0.062 (SE_a_)	0.032 ^n.s.^		No mediation
FoMO → SMSU	0.599 (b)	0.041 (SE_b_)			
MT → SMSU				0.011 * (*t* = 2.547)	
RT → FoMO	0.279 (a)	0.070 (SE_a_)	3.878 ***		Partial mediation
FoMO → SMCU	0.620 (b)	0.037 (SE_b_)			
RT → SMCU				0.009 ** (*t* = 2.624)	
RT → FoMO	0.279 (a)	0.070 (SE_a_)	3.845 ***		Full Mediation
FoMO → SMSU	0.599 (b)	0.041 (SE_b_)			
RT → SMSU				0.194 ^n.s.^ (*t* = 1.300)	
SCO → FoMO	0.278 (a)	0.064 (SE_a_)	4.205 ***		Full Mediation
FoMO → SMCU	0.620 (b)	0.037 (SE_b_)			
SCO → SMCU				0.374 ^n.s.^ (*t* = 0.889)	
SCO → FoMO	0.278 (a)	0.064 (SE_a_)	4.164 ***		Partial mediation
FoMO → SMSU	0.599 (b)	0.041 (SE_b_)			
SCO → SMSU				0.000 *** (*t* = 3.901)	

Remark: 1. MT = maximizing tendency; RT = regret tendency; SCO = social comparison orientation; FoMO = fear of missing out; SMCU = social media compulsive use; SMSU = social media surveillance use. 2. Z = ab/SE_ab_; SE_ab_ = √(a^2^ SE_a_^2^ + b^2^ SE_b_^2^). * *p* < 0.05; ** *p* < 0.01; *** *p* < 0.001; n.s. not significant.

## Data Availability

Data are available upon request.
